# It Takes Two to Tango: Coupling of Angiogenesis and Osteogenesis for Bone Regeneration

**DOI:** 10.3389/fbioe.2017.00068

**Published:** 2017-11-03

**Authors:** Andrea Grosso, Maximilian G. Burger, Alexander Lunger, Dirk J. Schaefer, Andrea Banfi, Nunzia Di Maggio

**Affiliations:** ^1^Department of Biomedicine, University Hospital, University of Basel, Basel, Switzerland; ^2^Department of Plastic, Reconstructive, Aesthetic and Hand Surgery, University Hospital of Basel, Basel, Switzerland

**Keywords:** angiogenesis, bone and bones, vascular endothelial growth factor, biomaterials, regenerative medicine, bone tissue engineering

## Abstract

Bone regeneration is a complex process requiring highly orchestrated interactions between different cells and signals to form new mineralized tissue. Blood vessels serve as a structural template, around which bone development takes place, and also bring together the key elements for bone homeostasis into the osteogenic microenvironment, including minerals, growth factors and osteogenic progenitor cells. Vascular endothelial growth factor (VEGF) is the master regulator of vascular growth and it is required for effective coupling of angiogenesis and osteogenesis during both skeletal development and postnatal bone repair. Here, we will review the current state of knowledge on the molecular cross-talk between angiogenesis and osteogenesis. In particular, we will focus on the role of VEGF in coupling these two processes and how VEGF dose can control the outcome, addressing in particular: (1) the direct influence of VEGF on osteogenic differentiation of mesenchymal progenitors; (2) the angiocrine functions of endothelium to regulate osteoprogenitors; (3) the role of immune cells, e.g., myeloid cells and osteoclast precursors, recruited by VEGF to the osteogenic microenvironment. Finally, we will discuss emerging strategies, based on the current biological understanding, to ensure rapid vascularization and efficient bone formation in regenerative medicine.

## Introduction

Bone regeneration entails a complex series of biological events, with the interplay of different cell types and the orchestration of several intracellular and extracellular signaling pathways. Bone health requires vascular control since blood vessels are key regulators for bone homeostasis, both providing nutrients and minerals and serving as structural templates for bone development (Hankenson et al., 2011). In the bone marrow, the vasculature also provides a niche environment for hematopoietic stem cells (HSCs), regulating their quiescence and mobilization. HSC and progenitors have been found in the proximity of small arterioles and specialized sinusoids (Kunisaki et al., 2013), where different cell types, including endothelial cells, perycites, stromal progenitors and sympathetic neuronal cells, contribute to the maintenance of HSC self-renewal [for a comprehensive recent review, see Morrison and Scadden (2014)]. In addition to these well-known functions, blood vessels have been recently ascribed a so-called angiocrine function, i.e., providing paracrine signals that coordinate growth, differentiation, and regeneration of different tissues, including bone, where they can promote osteogenesis (Ramasamy et al., 2016). Therefore, angiogenesis and vascular cells can affect biological processes in the bone/marrow organ at several different levels.

**Figure 1:**
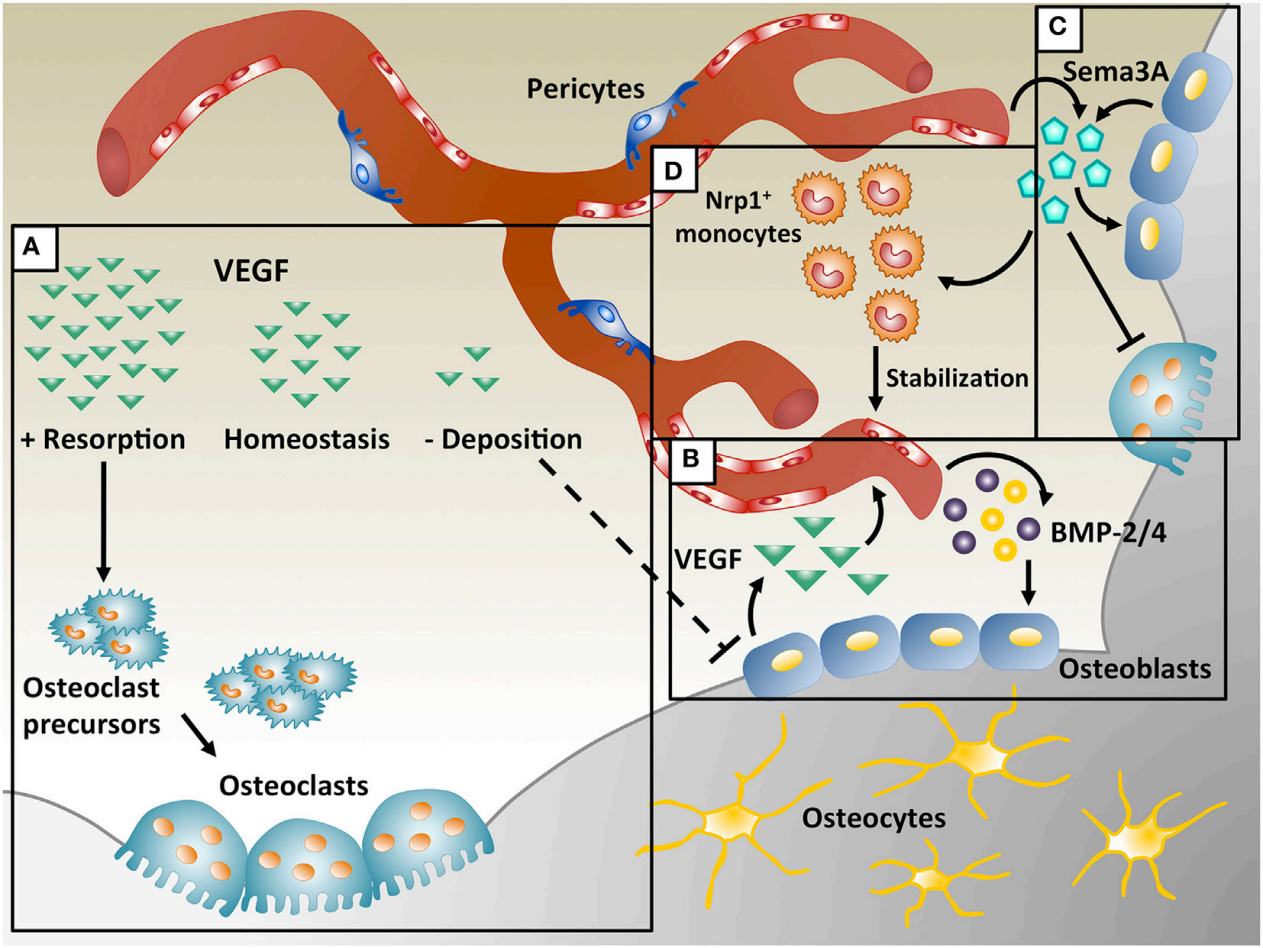
Coupling of angiogenesis and osteogenesis during intramembranous ossification. **(A)** Physiological levels of vascular endothelial growth factor (VEGF) maintain bone homeostasis, whereas too little VEGF interrupts osteoblast differentiation and too much VEGF increases osteoclast recruitment, leading to bone resorption. **(B)** During bone repair, VEGF is produced by osteoblasts and promotes migration and proliferation of endothelial cells. In turn, endothelial cells secrete osteogenic factors, like bone morphogenetic protein (BMP)-2 and BMP-4, which support osteoblast differentiation. **(C)** VEGF dose dependently regulates Sema3A expression in endothelial cells and Sema3A from different sources suppresses osteoclast differentiation and stimulates bone deposition. **(D)** Sema3A is also responsible for the recruitment of neuropilin 1-expressing (Nrp1^+^) monocytes, which promote vessel stabilization.

Vascular endothelial growth factor-A (VEGF) A is one of the most important regulators of angiogenesis and it is critical for both bone development and regeneration. In these processes VEGF has a dual role, acting both on endothelial cells to promote their migration and proliferation, and stimulating osteogenesis through the regulation of osteogenic growth factors (Schipani et al., 2009). VEGF is required for endochondral bone formation, where it promotes vessel invasion and recruitment of chondroclasts into hypertrophic cartilage, enabling the replacement of the cartilaginous template by bony callus (Gerber et al., 1999; Carlevaro et al., 2000; Hu and Olsen, 2016a), but also for intramembranous ossification (Street et al., 2002; Carvalho et al., 2004; Wan et al., 2008; Percival and Richtsmeier, 2013). Angiogenesis and osteogenesis are, therefore, intimately connected and they must be tightly coupled for physiological bone function. In fact, alterations in vascular growth can compromise physiological bone healing, e.g., leading to osteonecrosis, osteoporosis, and non-union fractures (Dickson et al., 1994; Martinez et al., 2002; Feng et al., 2010; Fassbender et al., 2011; Kaushik et al., 2012; Zhao et al., 2012). On the other hand, VEGF has also been described to inhibit osteoblast differentiation and to compete with PDGF-BB for binding to PDGF-Rs, impairing pericyte function, leading to the formation of immature blood vessels and to the interruption of the coupling of angiogenesis and osteogenesis (Greenberg et al., 2008; Schonmeyr et al., 2010; Song et al., 2011). Moreover, VEGF overexpression may also cause bone resorption due to excessive osteoclast recruitment (Helmrich et al., 2013). These data suggest that VEGF can have opposite effects on bone physiology under different circumstances (Figure 1A), but the underlying mechanisms through which VEGF regulates bone homeostasis are not yet fully understood, posing a challenge to the design of rational therapies.

Vascularization also plays a crucial role in bone tissue engineering, which aims at developing bone substitutes to replace large tissue losses due to trauma, surgery, or other clinical conditions where physiological bone repair is insufficient. In fact, upon implantation *in vivo*, a major challenge for clinical-size bone substitutes is the maintenance of cell viability in the graft core, which critically depends on the rapid invasion by host blood vessels. Poor blood perfusion results in cell death due to lack of oxygen, nutrients, and waste removal. Furthermore, functionally perfused vascular networks also mediate the recruitment of osteoprogenitors, HSC and immune cells, which play important roles in tissue regeneration and remodeling. Although several different strategies to improve vascularization of osteogenic grafts are currently being investigated, success so far has been limited. Elucidation of the molecular cross-talk between angiogenesis and osteogenesis is needed to exploit the therapeutic potential of VEGF and to design strategies to improve both efficient vascularization and bone formation.

## Coupling of Angiogenesis and Osteogenesis by VEGF During Intramembranous Ossification

Intramembranous ossification underlies the development of craniofacial bones and also the generation of tissue-engineered bone grafts, and it strongly relies on coupling of osteogenesis and angiogenesis. Contrary to endochondral ossification, in which bone develops through a cartilage intermediary, during intramembranous ossification progenitors directly differentiate into osteoblasts. Osteoprogenitors gather in the area of ossification and bone morphogenetic protein (BMP) signaling upregulates the transcription factor Runx2, which kickstarts the expression of specific proteins, like BSP, osteocalcin, and osteopontin, necessary to produce mineralized matrix (Huang et al., 2007).

Vascular endothelial growth factor has a crucial role in intramembranous ossification and its loss causes developmental deficits in skull bones and delayed healing during post-natal repair. Deletion of Vegf in Osterix-positive osteoblast precursors reduced calvarial and mandibular ossification (Wang et al., 2007; Hill et al., 2015; Duan et al., 2016). During intramembranous bone regeneration, exposure to hypoxia in the initial inflammatory phase stimulates osteoblasts to release several factors, including VEGF, *via* the HIF-1α pathway, inducing endothelial migration and proliferation and vessel permeability (Wang et al., 2007). The new vessels increase the supply of nutrients, oxygen and minerals necessary for osteogenesis and may recruit osteoprogenitors to the injury site. Furthermore, endothelial cells also produce osteogenic factors (e.g., BMP-2 and BMP-4) that promote osteoblast differentiation, while differentiating osteoblasts secrete angiogenic factors (e.g., PDGF-BB and VEGF) to further support angiogenesis by a positive feedback loop (Duan et al., 2015, 2016) (Figure 1B).

## Direct VEGF Effects on Osteogenic Differentiation of Mesenchymal Progenitors and Bone Regeneration

Some non-endothelial cells also express VEGF receptors, including osteoprogenitors, pericytes, and osteoclasts. Although osteoblast expression of VEGF receptors is variable, several reports indicate that VEGF can directly affect osteoblast differentiation maintaining postnatal bone homeostasis through autocrine and intracrine mechanisms. VEGF overexpression in human mesenchymal stromal/progenitor cells (MSCs) increased the deposition of mineralized extracellular matrix (ECM), while overexpression of sFLT-1, a secreted blocker of VEGF, reduced mineralization (Mayer et al., 2005). Moreover, mice with deletions of VEGF receptors Vegfr1 or Vegfr2 in osteoblastic cells showed low bone density and reduced number of osteoprogenitors in the bone marrow, indicating that both receptors in osteoblastic cells are important for postnatal bone formation (Liu et al., 2012). As mentioned above, maturing osteoblasts are one of the main sources of VEGF during intramembranous bone formation. Hu and Olsen showed that at the injury sites of a cortical bone defect, osteogenic cells, including osteoprogenitors, pre-osteoblasts and mature osteoblasts, are important sources of VEGF and that VEGF deletion specifically in osteoblasts disrupts the coupling of angiogenesis and osteogenesis and delays the healing process (Hu and Olsen, 2016a) (Figures 1A,B). Although the best-characterized functions of VEGF require its secretion, intracellular VEGF has been recently described to control transcriptional regulation and cell survival also through intracrine signaling (Liu et al., 2011; Liu and Olsen, 2014). Osteoblast-specific and conditional VEGF knockout mice exhibited an osteoporosis-like phenotype, with decreased bone mass and increased bone marrow fat (Liu et al., 2012). Here VEGF acted as a regulator of stem cell fate: it stimulated osteoblastic and blocked adipogenic differentiation by an intracellular mechanism involving the transcription factors RUNX2 and PPARy2, rather than by paracrine signaling (Liu et al., 2012; Berendsen and Olsen, 2014, 2015). However, the precise mechanisms by which intracrine VEGF regulates osteoprogenitor fate are not yet fully understood.

## Osteogenesis Promotion by Endothelial Angiocrine Factors

The microvascular circulation has an important role in sustaining the homeostasis of resident stem cells and guiding the regeneration and repair of adult organs (Rafii et al., 2016). A recently described concept is that vascular cells, besides their role as building blocks of the nutrient transport network, may also regulate the function of tissue-specific cells in the vicinity of blood vessels in several organs, through the release of paracrine signals. Tissue-specific endothelial cells mastermind this complex task supplying stimulatory or inhibitory growth factors, morphogens, ECM components, and chemokines that are collectively defined as angiocrine factors (Rafii et al., 2016). In the skeletal system, blood vessels are heterogeneous and functionally specialized. Recent studies have divided bone capillaries in two main subtypes: type H vessels are mainly present in the metaphysis of long bones and are highly positive for CD31 and Endomucin (CD31^hi^Emcn^hi^); type L, which are an extension of type H vessels, form sinusoidal vessels within the hematopoietic bone cavity and are less strongly positive for CD31 and Endomucin (CD31^lo^Emcn^lo^). Type-H endothelium displays high proliferative activity and secretes factors that regulate osteoblast function and chondrocyte proliferation (Sivaraj and Adams, 2016; Langen et al., 2017). Furthermore, osteoprogenitor cells are selectively positioned around type-H, but not type-L, capillaries (Kusumbe et al., 2014).

Bone morphogenetic proteins have been described as one of the main classes of molecules regulated by VEGF in both osteoblasts and endothelial cells (Maes et al., 2010; Yang et al., 2013). For example, it has been reported that in conditional knockout mice, BMP-2 deletion specifically in osteoblasts caused reduction in both VEGF levels and microcapillaries in the bone marrow, together with MSC numbers and their ability to form CFU-f and CFU-O colonies (Yang et al., 2013). On the other hand, VEGF can activate endogenous BMP-2 expression in vessel-associated MSC through the activation of the Akt/β-catenin pathways (Maes et al., 2010). BMP-2 produced by osteoblasts acts in an autocrine manner and stimulates osteoblasts to differentiate, produce VEGF, and further increase BMP-2 protein expression (Yang et al., 2013).

On the other hand, VEGF can upregulate BMP expression also in endothelial cells (Figure 1B), inducing osteogenic differentiation and matrix mineralization. Interestingly, both inactivation and overexpression of Noggin, a secreted BMP antagonist, specifically in osteoblasts led to reduced bone mass, indicating the importance of Noggin levels during bone formation (Ramasamy et al., 2014). Expression of Noggin in endothelial cells is controlled by Notch signaling. Mutants lacking Notch activity specifically in endothelial cells exhibited a significant reduction in Noggin expression, which impaired osteogenesis and chondrocyte differentiation through its angiocrine function. Moreover, angiogenesis defects induced by decreased VEGF expression in chondrocytes could be prevented by Noggin administration. Therefore, the cross-talk between the endothelium and other cell types ensures the coupling of angiogenesis and bone formation in the skeletal system (Ramasamy et al., 2014).

Increasing evidence suggests that numerous other factors in addition to BMPs regulate bone remodeling, as well as bone development and repair. VEGF appears to have a central role during these processes and its cross-talk with other factors needs to be elucidated. The Semaphorin (Sema) class of molecules has been described to both regulate VEGF-induced angiogenesis and bone homeostasis. The Semaphorin family includes eight subclasses of glycoproteins originally described as axon guidance molecules during neuronal development (Kolodkin et al., 1993; Luo et al., 1993). Semaphorins have been also involved in several other biological processes, including bone biology, angiogenesis, cancer progression, and immune disorders (Behar et al., 1996; Miao et al., 1999; Roth et al., 2009; Chaudhary et al., 2014; McKenna et al., 2014). In particular, both Sema3A and VEGF share signaling through neuropilin 1 (Nrp1). In fact, Nrp1 is a coreceptor for the VEGF_165_ isoform to regulate endothelial cell migration during angiogenesis *via* VEGFR2 and it is also essential for VEGF-induced vascular permeability and arteriogenesis. On the other hand, recent studies have shown the importance of Sema3A in the regulation of bone homeostasis (Figure 1C). Hayashi et al. showed that Sema3A has an osteoprotective effect by both suppressing osteoclast bone resorption and increasing osteoblastic bone formation, as evidenced by the severe osteopenic phenotype of Sema3A knock-out mice (Hayashi et al., 2012; Fukuda et al., 2013). Mechanistically, Sema3A activates the canonical Wnt/β-catenin pathway in the process of osteoblast differentiation and suppresses macrophage-colony-stimulating factor (M-CSF)-induced osteoclast differentiation through the Rho A signaling pathway (Hayashi et al., 2012). Interestingly, more recently it has been found that VEGF can dose dependently inhibit endothelial expression of Sema3A in skeletal muscle (Groppa et al., 2015), suggesting the possibility that dysregulation of Sema3A expression by VEGF delivery could also affect bone formation.

## Recruitment of Osteoclasts and Immune Cells by VEGF

Vascular endothelial growth factor is not only a key inducer of endothelial proliferation and vascular growth but also has direct and indirect effects on bone development by affecting various cell types involved in the process. MSC, osteoprogenitors, osteoblast, and osteoclasts all express both VEGF and VEGF receptors and respond to VEGF signaling by increased recruitment, differentiation, and activity (Dirckx et al., 2013). Moreover, VEGF also recruits different populations of monocytes and macrophages (Barleon et al., 1996). In the inflammatory phase of physiological bone repair, VEGF expression is induced by hypoxia in osteogenic cells (Street et al., 2000), leading to the recruitment of immune cells. In fact, mice lacking *vegf* expression by osteoblasts have decreased macrophages in a tibia cortical defect as well as reduced vascular density (Hu and Olsen, 2016a). Furthermore, VEGF also has a role in osteoclast function: M-CSF, receptor activator of nuclear factor kappa-B ligand, and VEGF are necessary for recruiting and programming osteoclast differentiation and they are released by both osteoblasts and endothelial cells (Kristensen et al., 2013). Osteopetrotic mice are deficient in osteoclasts, monocytes, and macrophages due to a mutation in M-CSF, but VEGF delivery increases osteoclastogenesis and bone resorption, while a VEGF antagonist suppresses this process (Niida et al., 1999). These data revealed for the first time that VEGF can substitute for M-CSF in osteoclast recruitment and differentiation. Recently, it has been shown that VEGF recruits osteoclast progenitors in arthritic joints through VEGFR-1 (Flt-1) and subsequent phosphorylation of focal adhesion kinase (Matsumoto et al., 2002). VEGF can also directly stimulate osteoclastic bone resorption and survival of mature osteoclasts *via* VEGFR-2 (Flk-1) (Nakagawa et al., 2000; Yang et al., 2008). Furthermore, VEGF overexpression by genetically modified bone marrow-derived MSC caused excessive osteoclast recruitment and bone resorption in tissue-engineered osteogenic constructs (Helmrich et al., 2013) (Figure 1A).

A specific population of monocytes coexpressing CD11b and Nrp1, named neuropilin-expressing monocytes (NEMs), promote smooth muscle cell recruitment and arteriogenesis by TGF-β1 and PDGF-BB secretion during VEGF-induced angiogenesis (Zacchigna et al., 2008) and also accelerate vascular stabilization, i.e., the ability of newly induced vessels to persist independently of further VEGF stimulation (Groppa et al., 2015). It has been recently shown that Sema3A is specifically responsible for NEM recruitment (Figure 1D) and that VEGF dose dependently inhibits vessel stabilization by impairing both endothelial Sema3A expression and NEM recruitment, leading to decreased TGF-β1 and endothelial SMAD2/3 activation (Groppa et al., 2015). High VEGF doses also have an antipericyte effect by competing with PDGF-BB for PDGF-Rβ binding (Greenberg et al., 2008). Control of VEGF dose is therefore crucial both for vessel stabilization and pericyte coverage, both of which have been shown to be important for osteogenesis/angiogenesis coupling (Hu and Olsen, 2016b).

## Strategies for Rapid Vascularization and Efficient Bone Formation in Regenerative Medicine

The equilibrium between VEGF-triggered angiogenesis, osteogenesis and bone resorption is clearly key to engineer vascularized bone. The design of rational strategies, to fully exploit the potency of VEGF as an angiogenic regulator and at the same time to ensure robust bone formation, should take in consideration this complex cross-talk. Several clinical conditions require replacement of the damaged or lost bone tissue due to trauma or surgery, or also in idiopathic conditions such as avascular necrosis of the lunate, scaphoid, and talus bones, where endogenous bone repair is insufficient. Several regenerative medicine approaches have been investigated to restore vascularization and promote bone regeneration, including combinations of biomaterials with angiogenic growth factors and/or genetically modified progenitors. More recently, protein engineering approaches have enabled the generation of natural matrices decorated with bound growth factors that are presented to cells in their physiological context during bone repair (Martino et al., 2015). In fact, the effectiveness of most morphogens critically depends on their concentration in the microenvironment of target cells and the delivery profile is controlled by the total dose incorporated into the material, the kinetics of release and the stability of the protein. In most current strategies, release kinetics is defined by non-specific interactions between the protein and the material. However, boost release may lead to non-physiological, excessive microenvironmental doses, which in the case of VEGF promote the formation of aberrant and hyperpermeable vascular structures (Ozawa et al., 2004) and excessive osteoclast recruitment (Helmrich et al., 2013). Furthermore, after injury the clotting process generates a fibrin-based matrix rich in many growth factors (Bao et al., 2009), which is conducive to cell migration, morphogen presentation and progenitor differentiation. Therefore, current strategies aim at mimicking ECM embedding to reproduce physiological presentation of angiogenic signals within the bone defects. For example, a highly tunable fibrin-based platform has been recently optimized to precisely control the dose and duration of VEGF protein delivery in tissues (Sacchi et al., 2014). VEGF could be released only by enzymatic cleavage by invading cells *in vivo* and optimized delivery ensured normal, stable, and functional angiogenesis over a 500-fold dose range and improved perfusion of ischemic tissues. In a conceptually different strategy, a recombinant fibronectin fragment was engineered to contain the natural binding sites for fibrin, integrins, and growth factors (Martino et al., 2011). Delivery of this fragment within a fibrin construct together with BMP-2 and PDGF-BB significantly increased bone healing in a rat calvarial defect at very low and otherwise ineffective doses, thanks to their presentation in the physiological matrix context. Along similar lines, but with a reverse approach, engineering with a short domain of placenta growth factor-2 endowed any growth factor with superaffinity for ECM proteins (Martino et al., 2014). Such engineering of VEGF, PDGF-BB, and BMP-2 greatly improved both angiogenesis and bone formation in a calvarial defect. Therefore, biomaterials can be more than just carriers and can be engineered to reproduce an ECM-like environment decorated with growth factors, through either covalent or affinity-based interactions, that present physiological signals to endogenous promoters and promote bone healing.

## Conclusion

Vascular endothelial growth factor has a critical role in bone development and postnatal bone repair (Maharaj and D’Amore, 2007; Wilson et al., 2010; Liu et al., 2012). However, VEGF levels should be tightly controlled, since non-physiological doses may impair bone regeneration, directly affecting osteoblast differentiation and increasing bone resorption (Helmrich et al., 2013; Hu and Olsen, 2016a). Current studies of VEGF functions should aim at further elucidating the molecular crosstalk between angiogenesis and osteogenesis, clarifying the association between VEGF dose and bone function and VEGF effects on progenitor cells and bone matrix protein synthesis. This knowledge will provide rational bases for the design of novel therapeutic strategies to generate large-size vascularized bone grafts. To offer the basis for clinically applicable strategies, new approaches should also offer safety and regulatory advantages, such as providing “off-the-shelf” products, avoidance of genetic modification of implanted cells, and controlled duration of factor delivery.

## Author Contributions

AG, AB, and NDM wrote and revised the manuscript. MGB and AL wrote the manuscript. DJS revised the manuscript.

## Conflict of Interest Statement

The authors declare that the research was conducted in the absence of any commercial or financial relationships that could be construed as a potential conflict of interest.
